# Probing the Nanoscale Heterogeneous Mixing in a High-Performance Polymer Blend

**DOI:** 10.3390/polym14010192

**Published:** 2022-01-04

**Authors:** Alexander Paul Fellows, Debashis Puhan, Janet S. S. Wong, Michael T. L. Casford, Paul B. Davies

**Affiliations:** 1 Yusuf Hamied Department of Chemistry, University of Cambridge, Lensfield Road, Cambridge CB2 1EW, UK; apf36@cam.ac.uk (A.P.F.); mtlc2@cam.ac.uk (M.T.L.C.); pbd2@cam.ac.uk (P.B.D.); 2 Department of Mechanical Engineering, Imperial College London, Exhibition Road, London SW7 2AZ, UK

**Keywords:** polyetheretherketone, polybenzimidazole, high performance polymers, atomic force microscopy, nanoscale thermal analysis, infrared nanospectroscopy

## Abstract

The blend of polyetheretherketone (PEEK) and polybenzimidazole (PBI) produces a high-performance blend (PPB) that is a potential replacement material in several industries due to its high temperature stability and desirable tribological properties. Understanding the nanoscale structure and interface of the two domains of the blend is critical for elucidating the origin of these desirable properties. Whilst achieving the physical characterisation of the domain structures is relatively uncomplicated, the elucidation of structures at the interface presents a significant experimental challenge. In this work, we combine atomic force microscopy (AFM) with an IR laser (AFM-IR) and thermal cantilever probes (nanoTA) to gain insights into the chemical heterogeneity and extent of mixing within the blend structure for the first time. The AFM-IR and nanoTA measurements show that domains in the blend are compositionally different from those of the pure PEEK and PBI polymers, with significant variations observed in a transition region several microns wide in proximity to domain boundary. This strongly points to physical mixing of the two components on a molecular scale at the interface. The versatility intrinsic to the combined methodology employed in this work provides nano- and microscale chemical information that can be used to understand the link between properties of different length scales across a wide range of materials.

## 1. Introduction

High-performance polymers (HPPs) are polymers that exhibit desirable mechanical, and thermal properties even under harsh conditions. Polybenzimidazole (PBI) is a highly stable linear heterocyclic polymer characterised by its high strength, a low coefficient of thermal expansion, a thermal decomposition temperature greater than 500 °C, and broad chemical resistance [1,2]. However, there are issues with the processability of PBI products due to its high melt viscosity [3]. Polyetheretherketone (PEEK), on the other hand, is a melt processable thermoplastic that has been highly used in HPP engineering applications [4]. A polymer blend is a simple mechanical mixture of two polymers. The blend of PEEK and PBI combines the superior mechanical properties and thermal resistance of PBI with the melt process ability of PEEK. This blend can be extruded and injection moulded into complex geometries, thereby offering an excellent replacement to metals and alloys for application in many industries to meet cost-efficiency and sustainability needs. The synergy between the two polymers within the blend results in improved properties compared to the neat polymers. For example, a 50:50 blend of PEEK and PBI has high thermal stability and is better in overall mechanical and tribological performance than both PEEK and PBI on their own [1,5,6,7]. This raises interesting fundamental questions regarding the specific nanostructure and composition of the blend, as this may explain the observed improvements in properties.

Previous studies on PEEK-PBI blends, using atomic force microscopy (AFM), observed two distinct domains of different mechanical properties [8,9]. This suggests that the blend consists of domains of pure PEEK and pure PBI with no significant mixing or interaction. This conclusion, however, is incompatible with observations that pure PBI and pure PEEK absorb significantly less water (0.40% and 0.45% by weight, respectively [10,11]) than their 50:50 blend (6.5% by weight [12]). This indicates that at least some change in physical or chemical structure must be occurring within the blend, allowing for higher water absorption. However, no studies have been conducted that probe the causes of increased uptake of water in the blend.

PBI and PEEK are generally considered immiscible. If PBI and PEEK interact, the interaction is most likely to occur at the interface of the two domains and their compatibility can be increased by stimulation through tweaking the interfacial bonding [13,14]. However, understanding properties of the interface is extremely challenging, yet essential to an understanding on how a polymer blend interface could be designed to optimize the performance of a blend. Conventional AFM has been successfully applied to investigate the surface morphology and mechanical properties of many materials at sub-micron to nanoscale levels. It, however, cannot provide chemical information directly. Any chemical information of a material is inferred from the measured mechanical properties. Although separate spectroscopic or calorimetric techniques can be used in conjunction with AFM, they lack the spatial resolution to directly link with AFM measurements.

This article takes advantage of recent advances in AFM instrumentation that combines the nanoscale topographical probe from traditional AFM with measurements of thermal properties (nanoTA) or infrared spectra (AFM-IR) which have been used for decades to investigate the miscibility behaviour of various polymer systems [15,16]. Applying these hybrid techniques means that the thermal and chemical properties can be obtained and be directly compared to the physical and mechanical structure as observed through traditional AFM measurements [17,18,19]. The information obtained from these advanced AFM techniques has proven to be invaluable in many fields, from materials chemistry [20,21,22] to biophysics [23,24,25,26,27,28,29,30,31], where chemical mapping of nanoscale features has elucidated structures and processes that would have otherwise escaped detection. In this work, the nanoscale mixing of 50:50 PEEK-PBI blends was investigated for the first time using these novel techniques to gain better insight into its compositional heterogeneity. We examine the interface between domains in the blend focusing on: (1) variation in topographical and mechanical properties (probed by AFM), (2) changes in the chemical properties (probed by AFM-IR), and (3) differences in their thermal properties (probed by nanoTA).

## 2. Materials and Methods

### 2.1. Sample Preparation and Handling

Discs of the 50:50 PEEK-PBI blend (PPB) were donated by Hoerbiger America Inc, Houston, Texas, neat PEEK samples were provided by Victrex Plc, Lancashire, UK, and neat PBI polymer samples were provided by PBI Performance Products, Derby, UK. The polymer samples were cleaned with methanol, sectioned into ~200 nm thick slices using a diamond ultra-microtome, and mounted adhesive-free on clean glass slides for spectral and thermal analysis. It should be noted that some variations in thickness within a given section, as well as among microtomed, sections were unavoidable due to the elasticity of the materials and their resistance to shear. No pre-measurement heating of the samples was carried out. Samples were examined under dry nitrogen during testing to minimise spectral interference from atmospheric moisture.

### 2.2. AFM-IR and nanoTA Measurements

PEEK, PBI, and PPB sections were analysed by AFM-IR and nanoTA using a NanoIR2 (Anasys Instruments, Santa Barbara, CA, USA) equipped with a MIRcat laser system (Daylight Solutions, San Diego, CA, USA) containing four Quantum Cascade Lasers (QCLs) covering the 1125–2298 cm^−1^ spectral range. For AFM-IR measurements, gold-coated tip-cantilever assemblies (ATEC-CONTAu, Apex Probes, Bracknell, United Kingdom), with a 30 nm tip radius, spring constant 0.02–0.75 Nm^−1^, and resonant frequency 7–25 kHz, were used. IR measurements were performed in resonance-enhanced mode using the first harmonic cantilever resonance (~50–60 kHz) which was tracked using the Phase-Lock Loop (PLL). As the tip scanned an area at a scan rate of 0.5 Hz, an AFM-IR map was obtained by taking local IR spectra at positions 50–100 nm apart. These spectra were recorded with a spectral solution of 2 cm^−1^.

As the tip approaches the surface, the resonant frequency of the cantilever changes in response to any surface forces it experiences which can be modelled as a parallel spring-mass-damper system. Thus, the tip–surface coupling interactions were examined by monitoring the Lorentz contact resonance (CR) frequency of the cantilever as the tip was rastered across the surface.

In a nanoTA measurement, a heating voltage is applied to a stationary, tailored AFM tip. As the tip is heated, the material underneath (under pressure) also heats up and expands. The resulting vertical deflection of the cantilever is monitored against temperature and can be linked to the thermal properties of the material underneath—i.e., local thermal information can be obtained at a high spatial resolution. Note that events, such as melting, can alter the tip–sample interactions and thus change the vertical deflection profile. Therefore, changes in the gradient of the tip deflection-temperature plot, termed as thermal profile, are indicative of a thermal transition occurring in the underlying material. Tip–cantilever assemblies (ThermaLever Probes, Anasys Instruments, Santa Barbara, CA, USA) with a resistance range of 0.6–3.5 kΩ, resonant frequency between 55 and 80 kHz, and spring constant between 0.5 and 3 Nm^−1^ were used. The relationship between applied probe voltages and tip temperature was calibrated using a quadratic fit to the melting temperatures (T_m_) of three polymer calibration samples: polycaprolactone (PCL), polyethylene (PE), and polyethylene terephthalate (PET), with a T_m_ of 55, 116, and 235 °C, respectively. Thermal ramps were recorded with a heating rate of 1 °C/s and cooling rate of 100 °C/s, ranging between 35 °C and 350 °C with a temporal resolution of 150 data points per second, whilst using a triggered deflection termination of 0.25 V (peak voltage). The effect on the thermal profiles of previously applied thermal ramps at points at close proximity was appraised by varying the lateral spacing of the thermal ramps from ~2 μm down to ~200 nm. It was found that spacings of up to ~500 nm showed changes to the thermal profiles from previous measurements. Consequently, all thermal profiles were obtained at positions at least 1 μm from each other—i.e., the achievable spatial resolution is ~1 μm. Assuming the thermal property of the materials is isotropic, the effective penetration depth of the thermal probes is also around 500 nm. As the samples used were approximately 200 nm thick, this means thermal profile obtained received contributions from the whole thickness of the microtomed sample (albeit with a steadily diminishing contribution with depth) and depending on the sample thickness, potentially the underlying glass substrate. To ensure the observed features in the thermal profiles of the polymers are otherwise unaffected by the underlying glass, thermal profiles of ~10 mm thick polymer discs were also recorded for comparison. Maps of thermal properties were generated by obtaining 100 thermal profiles over 10 × 10 μm^2^ regions.

### 2.3. FTIR Spectra

**Figure A1:**
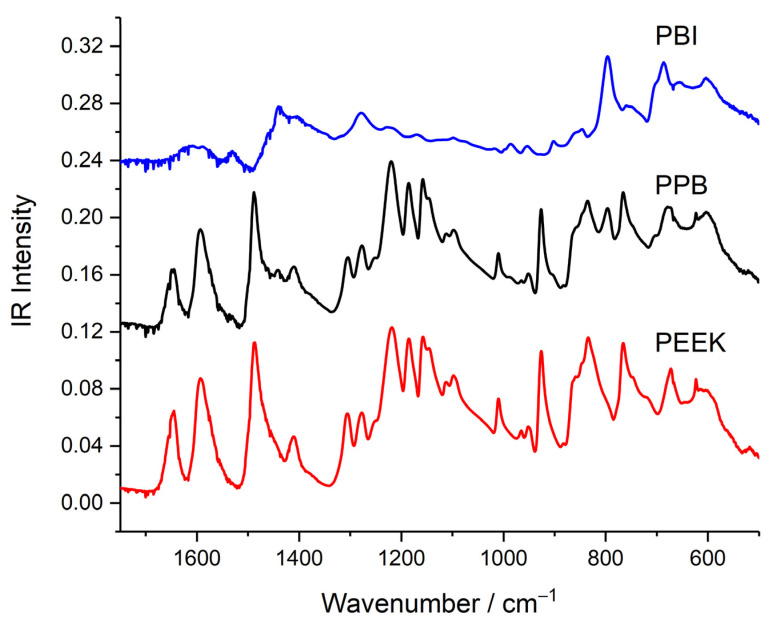
Bulk FTIR spectra of pure PBI and PEEK and their 50:50 blend, PPB, (spectra offset for clarity).

FTIR spectra were recorded on a Bruker Vertex V70 spectrometer in attenuated total-internal reflection (ATR) mode using a Specac Golden Gate accessory with a diamond ATR crystal and sapphire anvil cell. PEEK, PBI, and PPB samples in the form of swarf were obtained by cutting the polymer discs using a hacksaw and analysed under slight compression. Representative spectra of the polymer samples are shown in Appendix A, Figure A1 and band assignments based on literature values are given in Appendix A Table A1.

### 2.4. Similarity Index Calculations

Similarity indices are used to assess the similarities of IR spectra obtained at different regions within the PPB blend to those of pure PEEK and PBI. They are calculated using the residuals from normalised difference spectra relative to pure PEEK and PBI, as previously shown [32].Uncertainties are estimated based on the observed noise level and baseline deviations. This produces an index of unity for identical spectra and a vanishing index for spectra with no overlap. Since the spectra of PEEK and PBI (see Appendix A Figure A1) overlap, the similarity index between them is non-zero (0.77), as shown in Table 1. Thus, the similarity indices are used here for a qualitative assessment.

## 3. Results and Discussion

### 3.1. AFM and Infrared Nanospectroscopy

**Figure 1:**
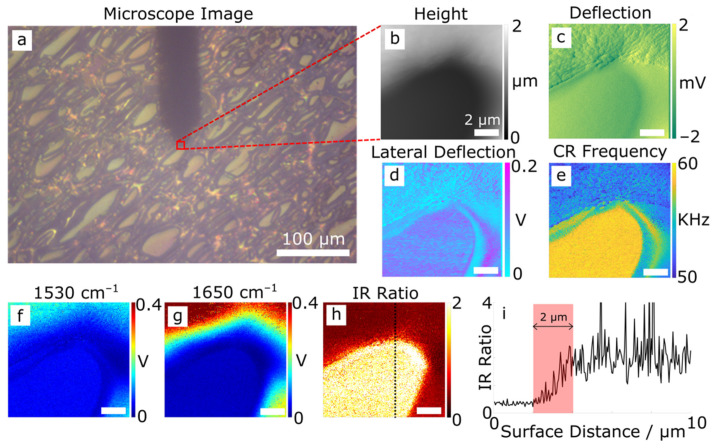
AFM-IR analysis of a microtomed section of PPB showing (**a**) an optical microscope image of the surface (including the AFM cantilever silhouette), as well as 10 × 10 μm maps of (**b**) height, (**c**) (vertical) deflection, (**d**) lateral deflection (friction), (**e**) contact resonance (CR) frequency, and IR intensity maps at (**f**) 1530 and (**g**) 1650 cm^−1^. A map of the ratio of the intensity at 1530 to that at 1650 cm^−1^ is shown in (**h**) and a corresponding vertical line profile through the ratio map traversing the domain boundary (dotted line in h) is shown in (**i**).

Microtomed sections of the PPB blend were examined with AFM and AFM-IR simultaneously. An optical microscope image of the sample is shown in Figure 1a, showing its domain structure, and identifying a 10 × 10 μm region mapped by AFM covering two domains and their boundary. The two domains are clearly identified in the AFM height map (Figure 1b) as having different heights, where the dark region is lower than the light region. Furthermore, the corresponding AFM vertical deflection map (Figure 1c) shows that the ‘low’ domain is smoother. The differences in height and topography of the two domains reflect their different responses to the microtome sectioning process. It is likely that the ‘low’ (dark) domains are stiffer; and undergo brittle failure. In contrast, the ‘high’ (light) domains are compressed during the cutting action, which subsequently recover after cutting. The lateral deflection map in Figure 1d reflects the lateral force the tip experiences as it scans the surface and is linked to the friction between the tip and the surface. It reveals a greater average coefficient of friction (high voltage) of the ‘low’ domain cf. the ‘high’ domain. Additionally, the ‘low’ domains also possess higher contact resonant (CR) frequency, see Figure 1e, confirming that it is stiffer than the ‘high’ domains. Considering the known elastic moduli of PBI (5.9 GPa) and PEEK (3.6 GPa) [9,10,11], along with their tribological characteristics [7,9,33], it is reasonable to conclude that the ‘low’ domains are more ‘PBI-like’, and the ‘high’ domains more ‘PEEK-like’. This observation is consistent with previous studies [8].

AFM-IR maps in Figure 1f and g show IR absorption intensities at 1530 and 1650 cm^−1^, $I_{1530}$ and $I_{1650}$, respectively. $I_{1530}$ corresponds to the in-plane ring vibration characteristic of 2-substituted benzimidazoles from PBI, and $I_{1650}$ the conjugated ketone C=O stretch from PEEK [1,34,35]. Both maps show greater intensity from the ‘high’ domain cf. the ‘low’ domain. This is likely due to the large height difference between the two domains (see Figure 1b), although contributions from potential tip–sample coupling artefacts, and/or differences in the coefficients of thermal expansion are plausible. These effects are removed by mapping $I_{1530}/I_{1650}$, as shown in Figure 1h, which identifies the ‘low’ domain as having a higher relative contribution from the 1530 cm^−1^ band cf. the 1650 cm^−1^ band and vice versa for the ‘high’ domain. This confirms the ‘low’ domains to be more ‘PBI-like’ and the ‘high’ domains more ‘PEEK-like’. From now on they are referred to in the manuscript as PBI-like and PEEK-like domains.

An important feature of the $I_{1530}/I_{1650}$ ratio map shown in Figure 1h is that the domain boundary is not sharply defined. Figure 1i presents $I_{1530}/I_{1650}$ taken along the dotted line indicated in Figure 1h. It shows that the transition between the two domains is gradual, and the width of the transition zone is ~2 μm. This suggests that polymer mixing occurs at a micron- or smaller length scales. It is also possible that the two polymers have reacted to form a new product at the interface. These possibilities will be further discussed below.

**Figure 2:**
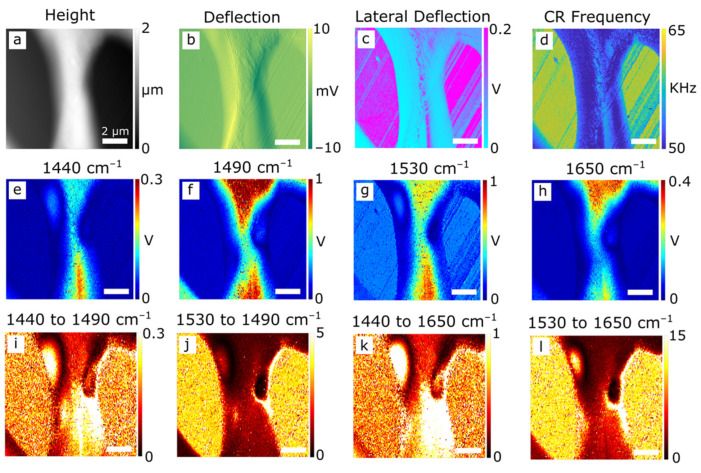
AFM-IR analysis of a PPB section showing 10 × 10 μm maps of (**a**) height, (**b**) vertical deflection, (**c**) lateral deflection, (**d**) CR frequency of the cantilever, and IR intensities at (**e**) 1440, (**f**) 1490, (**g**) 1530, and (**h**) 1650 cm^−1^, corresponding to known resonances of PEEK (1490 and 1650 cm^−1^) and PBI (1440 and 1530 cm^−1^). Ratio maps for each pair of PEEK-PBI bands are shown in (**i**–**l**).

**Figure A2:**
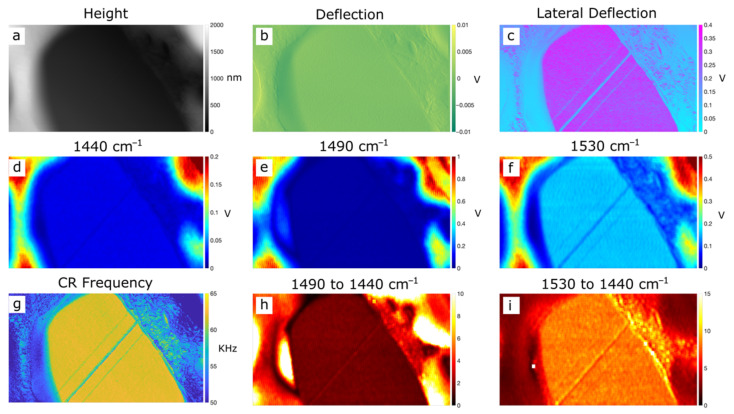
AFM-IR images of a microtomed section of PPB showing maps of: (**a**) height, (**b**) (vertical) deflection, (**c**) lateral deflection (friction), (**d**) IR intensity at 1440 cm^−1^ (PBI), (**e**) IR intensity at 1490 cm^−1^ (PEEK), (**f**) IR intensity at 1530 cm^−1^ (PBI), (**g**) contact resonance (CR) frequency, and IR intensity ratio maps of (**h**) 1490 and (**i**) 1530 cm^−1^, relative to 1440 cm^−1^.

AFM-IR maps of another region in the PPB are shown in Figure 2. Two ‘low’ domains are captured along with the ‘high’ domain region between them. The variations in height, relative smoothness, and CR frequency (Figure 2a–d) point to the same conclusions as in Figure 1. IR maps of specific frequencies: two related to PBI (1440 cm^−1^ from the in-plane benzimidazole deformation, and 1530 cm^−1^ from the 2-substituted benzimidazole in-plane ring stretching mode [34,35]), and two from PEEK (1490 cm^−1^ arising from the in-plane skeletal aromatic ring vibration, and 1650 cm^−1^ from the conjugated ketone carbonyl stretch), are shown in Figure 2e–h. The IR intensity ratios of PBI-related peaks to PEEK-related peaks, presented in Figure 2i–l, again confirm the low and high domains are PBI- and PEEK-rich respectively; with a gradual transition zone between them. AFM-IR maps of a further example of the transition region can be found in Appendix A Figure A2.

**Figure 3:**
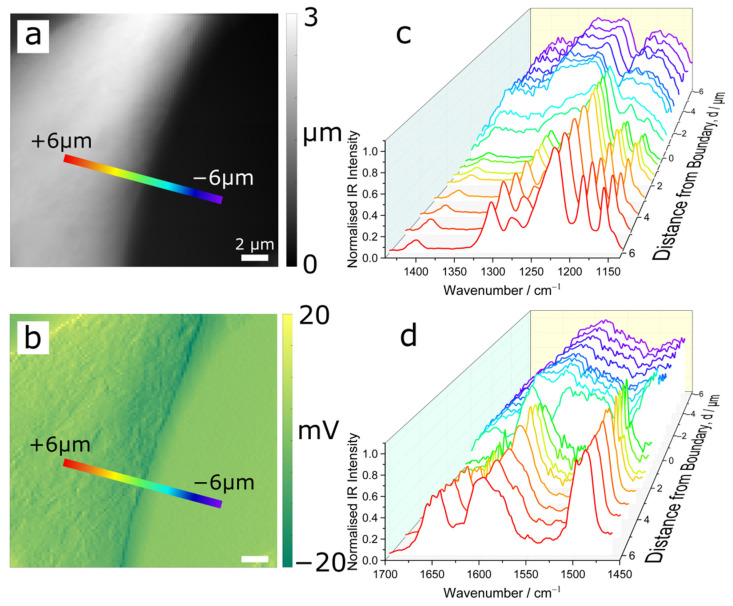
AFM-IR analysis traversing a PPB domain boundary, ‘low’ domain to the right, ‘high’ to the left. (**a**) AFM topography map, (**b**) AFM vertical deflection map, (**c**) AFM-IR spectra in the 1134–1440 cm^−1^ region, and (**d**) AFM-IR spectra in the 1460–1700 cm^−1^ region. The spectra were recorded at the positions shown in the maps in (**a**,**b**), where the ‘edge’ was determined from the vertical deflection plot (**b**) and negative distances correspond to the ‘low’ PBI domain side of the boundary. Spectra in the waterfall plots are normalised within each spectral region—i.e., dividing by the maximum intensity. The individual spectra are shown in Appendix A Figure A3.

**Figure A3:**
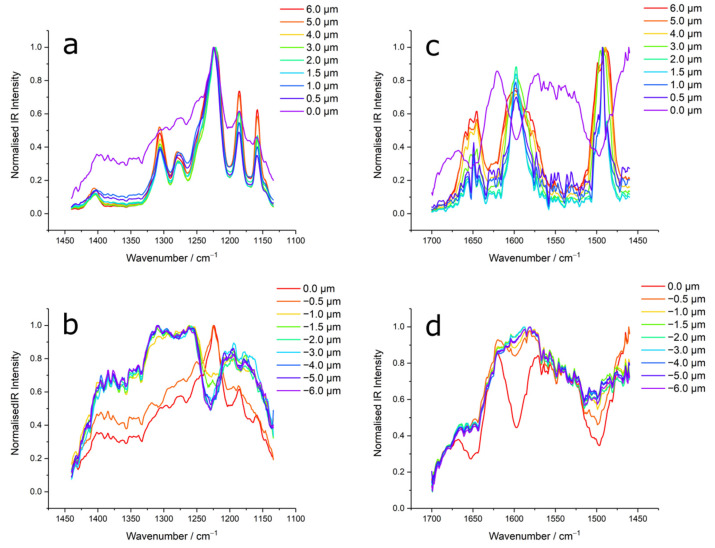
AFM-IR spectra of the PPB domains shown in Figure 4 overlapped to emphasize variations in the relative band intensity. The spectra in the ‘high’ domain are shown in (**a**,**c**) and those in the ‘low’ domain in (**b**,**d**).

Figure 3a,b show AFM height map and a vertical deflection map respectively of a representative domain boundary. Recall that the lighter shade (higher), rougher region is PEEK-like and the dark (lower), smoother region is PBI-like. AFM-IR point spectra were recorded along the path of the coloured bars, traversing the boundary from PEEK-like domain (red; distance from the edge, d > 0) to a PBI-like domain (purple; d < 0). Normalised AFM-IR spectra in the spectral range of 1134–1440 cm^−1^ and 1460–1700 cm^−1^, are presented in Figure 3c and d respectively (see also the associated overlapping spectra in Appendix A Figure A3). Spectra obtained within a domain, far from the boundary, reflect their expected identity and are relatively similar, i.e., chemically homogeneous. However, as the transition zone is approached, the spectra show gradual changes. These spectral changes are mostly in the form of relative band intensity variations rather than changes by means of the introduction or loss of resonances. As we approach the transition zone from the PEEK-like region (d from +6 mm to 0), the 1493 and 1598 cm^−1^ bands also show an appreciable decrease in their width (see also Figure A3c). Such changes are governed by intermolecular interactions and are indicative of a change in the local environment at the molecular level. The localised spectra show that most changes occur within a very narrow transition zone (~±1 μm), which agrees with the result in Figure 1i. It is noteworthy that the spectra at d = −0.5 μm, i.e., within the PBI-like domain, show strong bands characteristic of the PEEK-like domain spectra, e.g., the band at 1224 cm^−1^ (see Figure A3b). On closer inspection, the spectral features of PEEK are observed in the IR spectra obtained in PBI-like domain which subside moving away from the boundary. On the other hand, the effect of mixing is observed in the PEEK domain up to 6 μm from the boundary (Figure A3c).

**Figure 4:**
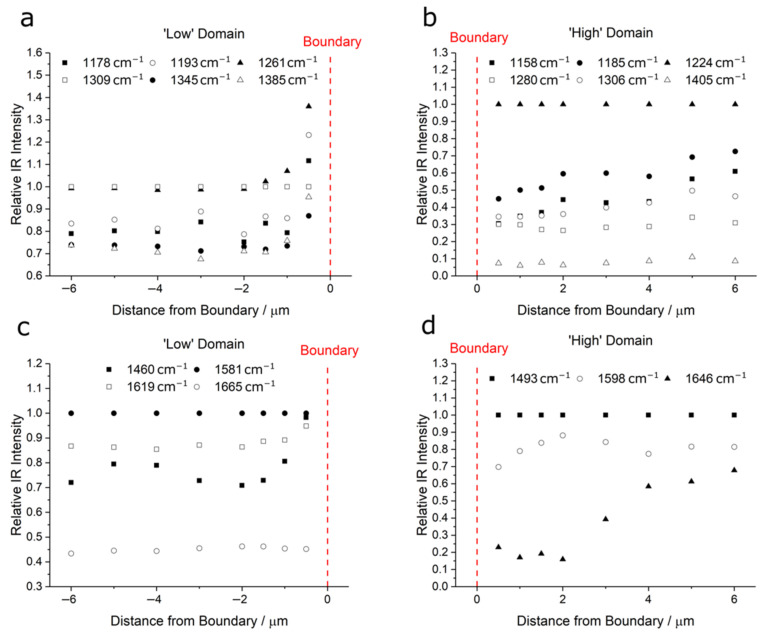
Analysis of the AFM-IR intensities of the PPB domains showing the relative intensities of the bands in the spectra presented in Figure 3 for the 1134–1440 cm^−1^ (**a**,**b**) and the 1460–1700 cm^−1^ (**c**,**d**) spectral regions within ‘low’ (**a**,**c**) and ‘high’ (**b**,**d**) domains. The intensities are normalised to the strongest band in the spectrum furthest from the boundary (i.e., d = −6 and d = +6 μm for the ‘low’ PBI and ‘high’ PEEK domains, respectively) within each of the two spectral regions investigated (each of which was covered by an individual QCL) i.e., to the band at 1309 cm^−1^ in (**a**), 1224 cm^−1^ in (**b**), 1581 cm^−1^ in (**c**), and 1493 cm^−1^ in (**d**).

Figure 4 quantifies the variation in relative band intensities with distance from the perceived domain boundary (defined using the AFM deflection map, d = 0) for both the 1134–1440 cm^−1^ spectral region (Figure 4a,b for PBI-like and PEEK-like domains, respectively) and the 1460–1700 cm^−1^ spectral region (Figure 4c,d for PBI-like and PEEK-like domains, respectively). All intensities are shown relative to the band with the highest intensity in the spectra taken furthest from the boundary (i.e., d = ±6 μm). Bands in these spectral regions from pure PEEK and pure PBI based on literature assignments are given in Table A1. In the ‘low’ PBI-like domains, relative intensities of the 1178, 1193, 1261, 1345, and 1385 cm^−1^ bands cf. the 1309 cm^−1^ band (Figure 4a) increase from d = −2 to 0 μm. For the ‘high’ PEEK-like domain bands, relative intensities of the 1158 cm^−1^, and 1185 cm^−1^ bands decrease gradually from d = 6 μm to d = 0, with more obvious decreases in the transition zone (Figure 4b).

**Figure A4:**
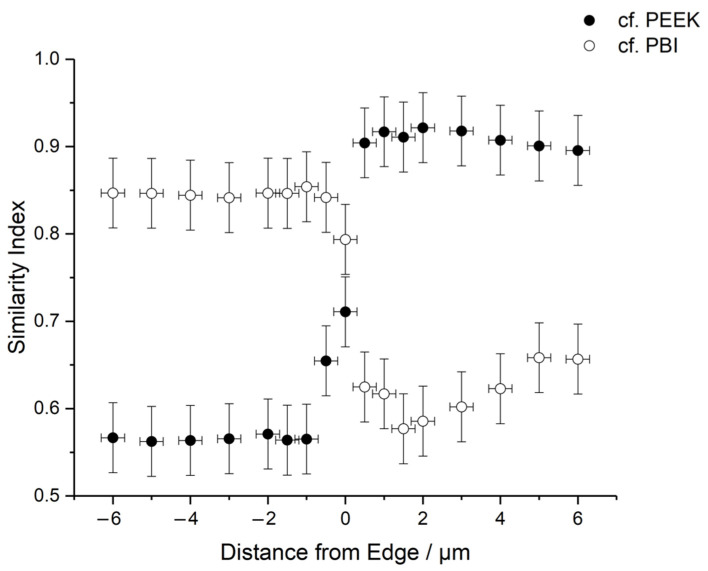
Combined similarity indices derived from both the 1134–1440 cm^−1^ and 1460–1700 cm^−1^ regions of the AFM-IR PPB spectra shown in Figure 4 and the AFM-IR spectra of pure PEEK and pure PBI.

Similar conclusions can be reached using peaks in the 1460–1700 cm^−1^ spectral range (Figure 4c,d) and is further supported by the similarity indices of these spectra, see Appendix A Figure A4. Although absolute concentrations cannot be inferred from similarity index calculations due to the measurement uncertainty and convolution of peaks in the spectra, qualitative changes are still indicative of structural similarity. The similarity indices in the transition region show the largest changes, consistent with spectral observations. Beyond this region (|d| > 2 μm), changes are only observed in the PEEK-like domains. This leads to two conclusions: firstly, that the level of mixing is greater within the PEEK-like domain. Secondly, the PBI-like domain consists mostly of PBI and it incorporates a similar distribution of PEEK through out the domain as there are no discernible changes to the spectra away from the transition region. In contrast, the ‘high’ PEEK domains have incorporated some PBI whose amount reduces with distance from the boundary. The mixing of the two polymers within each domain network disrupt the intermolecular environment of the PEEK and PBI molecules in their respective domains, resulting in alterations to their IR band ‘fingerprints’.

### 3.2. Thermal Nanomicroscopy

**Figure 5:**
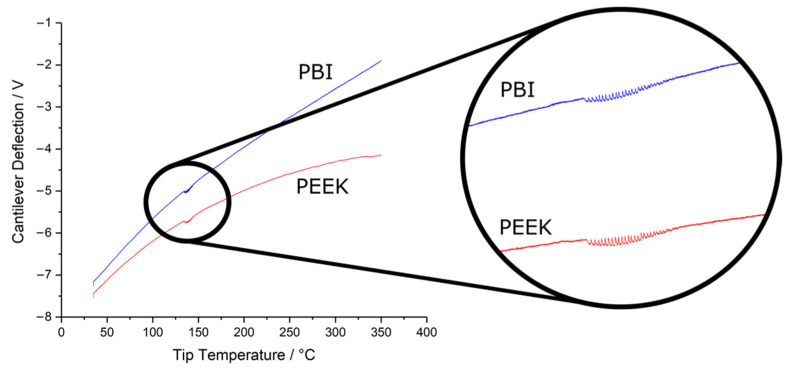
NanoTA analysis of microtomed sections of PEEK and PBI. Average temperature profiles of each material with the regions showing water release fringes expanded in the inset.

**Figure A5:**
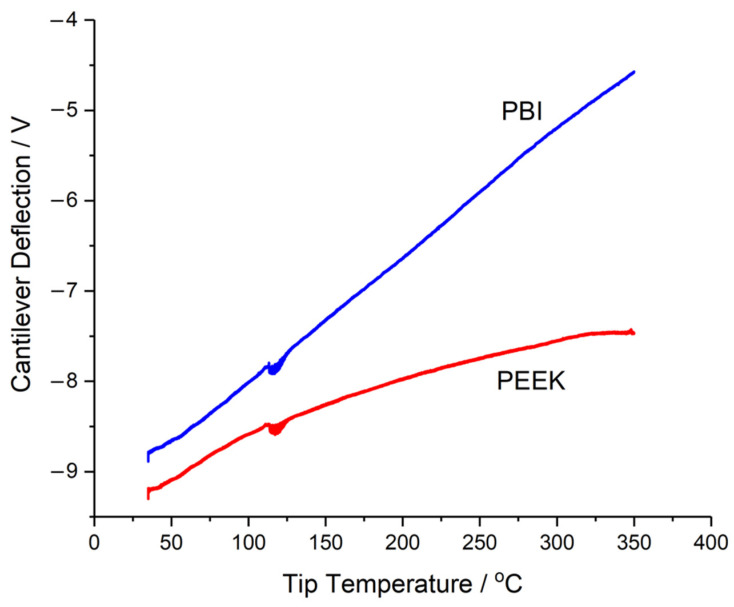
NanoTA profiles of 10 mm thick PBI and PEEK polymer samples.

The nanoTA profiles—which relates the deflection of the AFM cantilever due to the expansion of the surface underneath the AFM probe with increasing tip temperature—for pure PEEK and pure PBI are shown in Figure 5. Note that a nanoTA profile would reach a maximum during melting. While this is not observed in PEEK, its profile is plateauing at 350 °C, suggesting a melting transition is imminent. This agrees with the known melting point of PEEK at 343 °C. Under a fixed set of instrument parameters, the upward deflection of the cantilever will depend on several factors including the coefficient of thermal expansion, the thermal conductivity, the tip–material surface effective contact radius and the effective elastic modulus, all of which may be temperature-dependent. Furthermore, as the heat is applied from the tip, the temperature of the sample will decay with distance, resulting in an inherent penetration depth of the thermal analysis that is determined by the thermal properties of the material. Comparison of two profiles in Figure 5 shows that PBI has a higher rate of probe cantilever deflection than PEEK. This could result from differences in all the above factors and likely suggests that PBI has a higher heat penetration depth in comparison to PEEK. Furthermore, the increasing separation of the two profiles with temperature is indicative of greater thermal stability of PBI cf. PEEK. Since the samples used were 200 nm thick, nanoTA analysis was also conducted on 10 mm thick polymer samples to ensure that any contribution from the underlying glass substrate can be discounted. Thermal profiles for the 10 mm thick PBI and PEEK substrates yield similar deflection rates as ~200 nm thick samples (see Appendix A Figure A5), showing that contribution from the glass is negligible.

**Figure A6:**
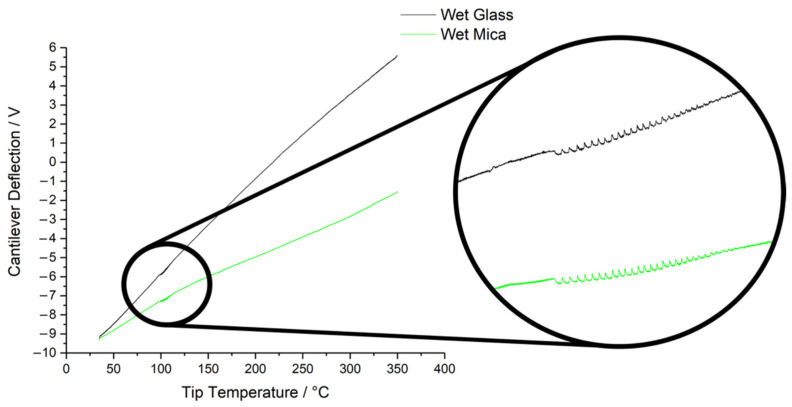
NanoTA profiles of wet glass and wet mica drawn from a clean LB trough showing the water deflection fringes at ~100 °C in the inset.

An interesting feature of the nanoTA profiles of PEEK and PBI are the set of fringes seen at ~130–140 °C (inset to Figure 5). These are attributed to the release of interstitial water, causing the cantilever to vibrate as the water evaporates. This phenomenon should not be compared with the boiling of bulk water since these interstitial water molecules are bound to the polymer molecular network and hence are exposed to an environment significantly different from that of bulk water [25]. This may influence the temperature at which the interstitial water is released. Analogous fringes are also observed on wet glass and mica surfaces (Appendix A Figure A6), but at ~100 °C because the boiling point of this surface water (i.e., not interstitial) is similar to that of bulk water. In contrast, fringes are absent from the profile of the extremely hydrophobic PE calibration sample, known to contain very little water. This confirms the fringes in Figure 5 originate from bound water. The fringes are also observed in thermal profiles of thick polymer samples, as shown in Appendix A Figure A5, which shows that they are inherent to the materials and do not appear from the underlying glass substrate.

The onset temperatures for water release, $T_{c-water}$, is defined as the lowest temperature at which the water fringes were observed in the NanoTA profiles, while $A_{w}$ is the difference in the area under the expected profile without the fringes (obtained by interpolation) and the actual profile. While $T_{c-water}$ of PBI and PEEK are indistinguishable, $\left|A_{w}\right|$ is greater for PEEK than for PBI. This suggests more interstitial water is present in PEEK which in turn causes larger forces on the tip–cantilever assembly on release and therefore increased changes in the cantilever deflection. This is unexpected given that both PEEK and PBI contain similar quantities of water under standard conditions [10,11] (and PBI is known to be more hygroscopic than PEEK under high temperature steam treatment [36]). It is, however, important to consider that the amount of water being released is likely to be highly dependent on the total material being probed (i.e., the effective thermal penetration depth). Hence, $A_{w}$ is not representative of the amount of interstitial water because the thermal penetration depth is highly influenced by the thermal properties of the material.

**Figure 6:**
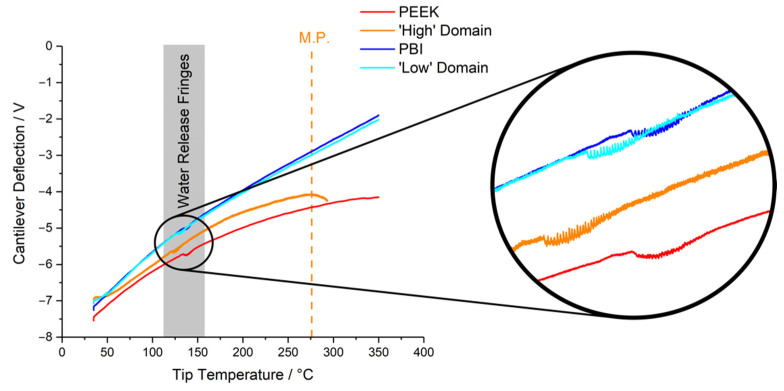
NanoTA deflection profiles for pure PEEK, pure PBI, and from the ‘high’ and ‘low’ domains of PPB, taken at least 5 μm distant from the boundary showing the regions of water release fringes in the inset.

NanoTA profiles of the two PPB domains obtained at least 5 mm from the domain boundary, alongside those of pure PEEK and pure PBI, are shown in Figure 6. The profiles for the ‘low’ PBI-like domains in PPB and pure PBI are practically indistinguishable, although the former shows a lower $T_{c-water}$. This means that their thermal properties are relatively similar over this temperature range despite the inclusion of PEEK in the ‘low’ PBI-like domain in PPB. The difference in $T_{c-water}$, however, shows that the molecular structure of the two materials must be different. The thermal profile of the ‘high’ PEEK-like domains and that of pure PEEK, however, have distinctive differences. Whilst they follow a similar trend initially, a clear melting transition of ‘high’ PEEK-like domains, shown as a maximum of its profile, is observed (see the dashed line in Figure 6), and is at a temperature well below the known melting point of PEEK of ~340 °C. In addition, $T_{c-water}$ of the ‘high’ PEEK-like domains in PPB is lower than that of pure PEEK (also indicated in Figure 6) which is indicative of structural differences. The results also indicate that PEEK chains in the PEEK-like domains of the blend experience a different molecular environment to that in pure PEEK due to the inclusion of PBI—i.e., the intermolecular forces experienced by these PEEK chains are reduced, lowering the melting point.

As mentioned earlier, the AFM-IR results (see Figure A4, also Figure 3) suggested that the similarity between ‘high’ PEEK domains in the PPB and pure PEEK (similarity index of 0.9) is greater than that between the ‘low’ PBI domains and pure PBI (similarity index of 0.85). In contrast, the nanoTA profiles suggest the opposite. It is important to note that these techniques are probing fundamentally different properties of the materials. Materials that have better thermal stability over the tested temperature range (i.e., PBI) would be expected to show smaller alterations to their thermal profiles due to minor changes to their structures (i.e., ‘low’ domains in PPB) than less thermally stable polymers (i.e., PEEK and the ‘high’ domains in PPB). On the other hand, small alterations to the structure of materials that affect intermolecular interactions can have a marked effect on their IR spectra, regardless of thermal stability.

**Figure 7:**
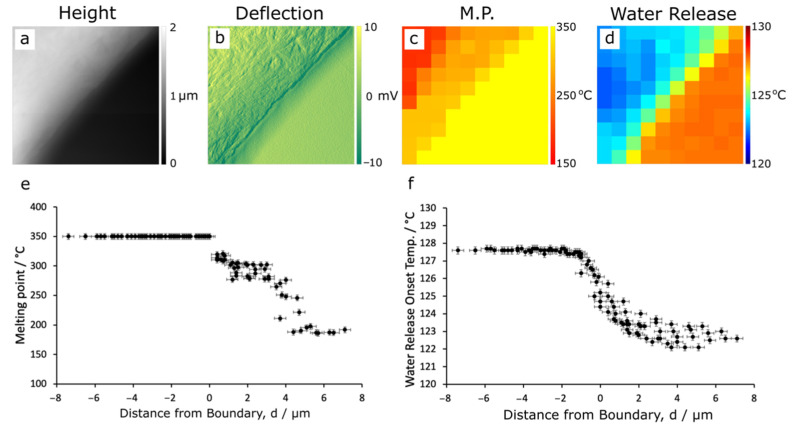
NanoTA analysis of PPB domains. (**a**) AFM height and (**b**) deflection maps. Corresponding microscopy images of the melting point transition (**c**) and water release onset temperature (**d**). Each pixel of these maps corresponds to 1 × 1 μm^2^. Changes in the melting points (**e**), and water release onset temperatures (**f**) are also shown with the distance from the approximate mid-point of the transition region calculated from the thermal microscopy images.

**Figure A7:**
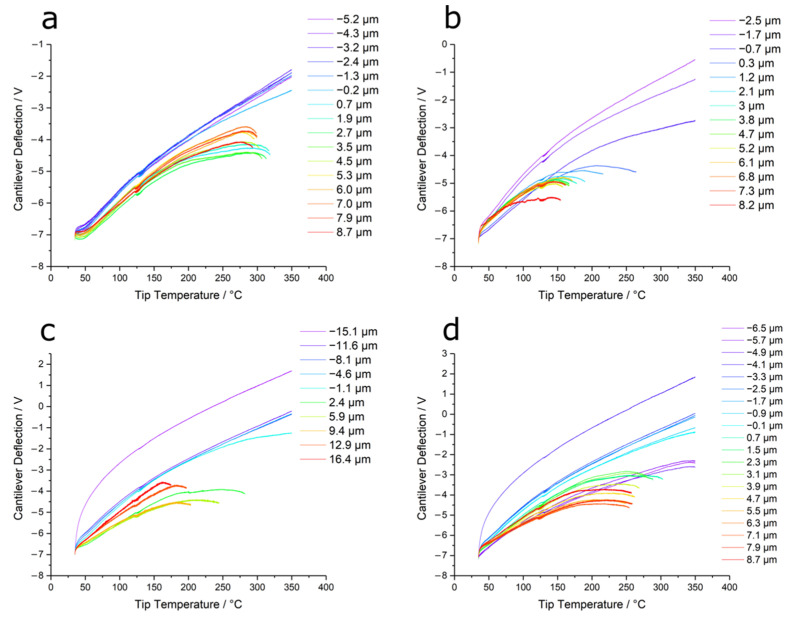
NanoTA temperature profiles of PPB traversing the domain boundary, with (**a**–**d**) corresponding to the domain boundaries shown in Figure A8(ai–aiv), respectively.

**Figure A8:**
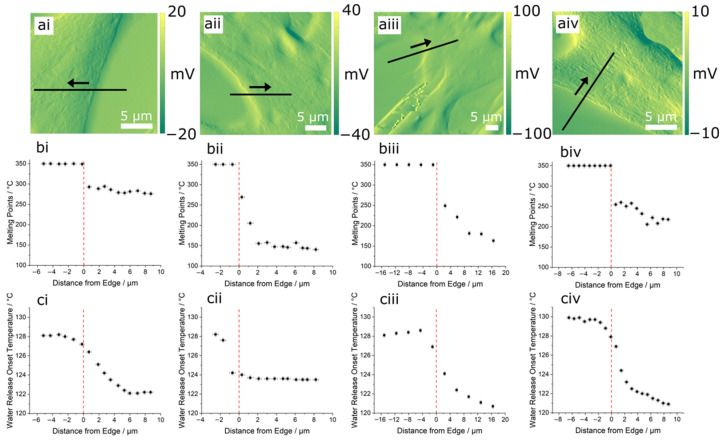
NanoTA analysis of PPB traversing the domain boundary. Each of the columns (**i**–**iv**) represents a different domain boundary. (**a**) AFM deflection maps where the solid black line denotes the locus of the sampling profile in (**b**,**c**) and the vertical dotted red line the position of the perceived domain boundary in the image. ‘Low’ and ‘high’ domains can be identified by the greater relative smoothness in the former, as previously indicated. (**b**) changes in the melting point (**c**) water release onset temperature. Uncertainties in the melting points and water release onset temperatures were estimated from uncertainties in determining the deflection maxima and fringe onset in the thermal profiles, respectively.

**Figure A9:**
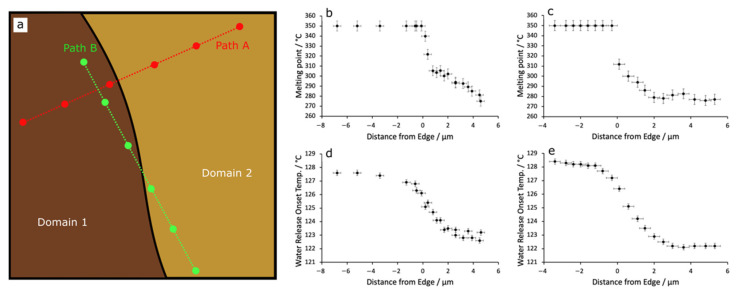
NanoTA analysis of PPB boundaries at high resolution showing (**a**) a schematic of two-line profile paths: path A (red) normal to the boundary, and path B (green) at a grazing angle, which achieves higher spatial resolution with respect to distance from the edge for a fixed separation of sampling points. Melting point transitions (**b**,**c**) and water release onset temperatures (**d**,**e**) for two different domain boundaries along path B are shown.

The thermal properties of PPB across the domain boundary are presented in Figure 7 where the AFM height (Figure 7a) and deflection (Figure 7b) maps are directly compared with the melting point (Figure 7c) and $T_{c-water}$ (Figure 7d) maps. Results from four other boundaries are shown in (Appendix A Figure A7 and Figure A8), recording line profiles close to perpendicular to the boundary (thus limiting the lateral resolution), as well as at four further boundaries (Appendix A Figure A9) where the line profiles were recorded close to tangential to the boundary (thus maximising the lateral resolution). The melting point and $T_{c-water}$ of each pixel is plotted against its shortest distance from the perceived boundary, shown in Figure 7e and f, respectively. The ‘low’ PBI-like domains (d < 0) do not melt and are assigned a melting point of 350 °C, i.e., the maximum accessible temperature. While in the ‘high’ PEEK-like domains, the melting temperature decreases as we move further into the domains (d > 0). $T_{c-water}$ also changes from high in PBI-like domains to low in PEEK-like domains. The changes observed in PEEK-like domains extend further from the boundary (from d = 0 to d = 6 µm), suggesting that more of the PBI polymer is embedded in the PEEK domain than PEEK polymer in the PBI-like domains where changes are observed from d = 0 to d = −2 µm. In all cases, $T_{c-water}$ of both PBI-like and PEEK-like domains are lower than those observed in the pure polymers (but slightly more so in the ‘high’ PEEK domains) indicating compositional changes throughout the PPB blend. The mixing process disrupts the polymer network and leads to a change in interactions between the polymer chains in the domain.

From the literature, the amount of water absorbed by PPB is 6.5% by weight [12] while it is 0.40% and 0.45% by weight in pure PBI and pure PEEK, respectively [10,11]. These results support substantial mixing of polymers at the interface and the width of the transition zone is in the range of microns. Mixing has changed the interactions between the polymer chains within domains which may lead to increased uptake of water.

## 4. Conclusions

In this work, the boundary between the observed domains of the 50-50 blend of PEEK and PBI has been investigated using AFM, AFM-IR, and nanoTA. AFM and AFM-IR mapping showed that the two domains can be distinguished topographically and spectroscopically. Physical properties, specifically the elasticity modulus, coefficient of friction, and surface roughness, as well as spectroscopic maps at known vibrational frequencies characteristic of PEEK and PBI, indicate that regions of the two domains away from the boundaries are similar to, but not identical with, those of the pure polymers. Local IR spectra showed a transition region of about 2–6 μm exists at the boundary of the two domains where gradual transitions of chemistry and thermal properties occur. Local nanoTA analysis traversing the domains verified the existence of a micron wide transition region. Within the range of temperature tested, the PBI-like domains did not undergo melting while the PEEK-like domains exhibited a lower melting point than pure PEEK. This indicated that the chain–chain interaction in the PEEK-like domains has been altered due to the infiltration of PBI into the PEEK polymer network. As a result, PPB blend absorbs more water than neat PEEK and neat PBI.

The present work investigates the mixing of PEEK and PBI beyond the bulk level mixing that gives the observed domain structure in the blend. Down to the nanoscale, it is shown that the mixing of PEEK and PBI leads to changes in interactions within the polymer network, which, in turn, affects the mechanical and thermal properties of polymer blends. Thus, understanding nanoscale mixing and associated structural changes provides critical insight into the macroscopic properties of PEEK–PBI blends and their performance in engineering applications. Moreover, hybrid techniques such as AFM-IR and nanoTA can be used to study and gain knowledge regarding how miscibility, phase separation, and water uptake of polymer systems is affected by polymer processing methods and parameters.

## Figures and Tables

**Table 1:** Similarity index calculations based on FTIR spectra of the bulk materials.

	PEEK	PBI	PPB
PEEK	1	-	-
PBI	0.77 ± 0.04	1	-
PPB	0.94 ± 0.03	0.83 ± 0.04	1

## Data Availability

The data presented in this study are available on request from the corresponding author.

## Appendix A

**Table A1:** Recorded FTIR band positions and literature assignments of bulk PEEK and PBI bands.

	Band/cm^−1^	Assignment
PEEK	1155	Aromatic in-plane C-H deformation
	1190	Asymmetric C-O-C stretching
	1215	Aromatic in-plane C-H deformation
	1277	Asymmetric C-O-C stretching
	1305	Ketone bend vibration
	1410	Skeletal ring vibration
	1485	Skeletal ring vibration
	1500	Skeletal ring vibration
	1593	Skeletal ring vibration
	1653	C=O Ketone stretching
PBI	1215–1235	In-plane C-H deformation
	1277–1287	Ring breathing mode
	1395–1528	In-plane ring deformation
	1590–1650	C=N/C=C stretching
